# Psychological distress, but not single-time endocrine stress markers, is associated with unexplained infertility: a prospective case–control study

**DOI:** 10.3389/fendo.2026.1859428

**Published:** 2026-06-26

**Authors:** Sertaç Ayçiçek, Berçem Ayçiçek

**Affiliations:** 1 Department of Obstetrics and Gynecology, Gazi Yaşargil Training and Research Hospital, Diyarbakır, Türkiye; 2 Department of Endocrinology and Metabolism, Istinye University Faculty of Medicine, Istanbul, Türkiye

**Keywords:** cortisol, DHEAS, HADS, Neuroendocrinology, psychological distress, Unexplained infertility

## Abstract

**Background:**

Psychological distress is common among women with unexplained infertility and may interact with neuroendocrine stress pathways. However, the relevance of single-time endocrine stress markers such as cortisol and dehydroepiandrosterone sulfate (DHEAS) in reflecting chronic emotional burden remains uncertain.

**Objective:**

To investigate the association between psychological distress and single-time endocrine stress markers in women with unexplained infertility and to compare psychometric assessment with synchronized hormonal measurements.

**Methods:**

In this prospective case–control study, consecutive women with primary unexplained infertility (n = 50) and age-matched fertile controls (n = 52) were enrolled from the same tertiary gynecology outpatient clinic. Psychological distress was assessed using the Hospital Anxiety and Depression Scale (HADS). Clinically relevant psychological distress was defined as HADS-Anxiety ≥8 and/or HADS-Depression ≥8. Serum cortisol and DHEAS concentrations were measured under standardized morning fasting conditions during the early follicular phase. Multivariable logistic regression analysis was performed using a parsimonious adjusted model including age, body mass index, smoking status, residential environment, cortisol level, and HADS-defined psychological distress.

**Results:**

Women with unexplained infertility demonstrated significantly higher HADS-Anxiety and HADS-Depression scores compared with fertile controls (p < 0.05 for both comparisons). Based on predefined HADS subscale cut-off values (HADS-A ≥8 and/or HADS-D ≥8), HADS-defined psychological distress was significantly more prevalent in the unexplained infertility group (76.0% vs. 42.3%, p = 0.001). Although follicle-stimulating hormone and estradiol levels differed significantly between groups, all measured values remained within physiological reference ranges. No statistically significant between-group differences were observed for cortisol, dehydroepiandrosterone sulfate (DHEAS), or anti-Müllerian hormone levels. In the final parsimonious multivariable logistic regression model adjusted for age, body mass index, smoking status, residential environment, cortisol level, and HADS-defined psychological distress, psychological distress remained independently associated with unexplained infertility (adjusted OR 3.907, 95% CI 1.610–9.478, p = 0.003). Exploratory ROC analyses demonstrated limited discriminatory performance of isolated single-time cortisol and DHEAS measurements for identifying HADS-defined psychological distress.

**Conclusion:**

Psychological distress was more prevalent among women with unexplained infertility, whereas single-time serum endocrine stress markers were not significantly different between groups. These findings support the relevance of psychological assessment in women with unexplained infertility; however, the cross-sectional case–control design does not allow causal inference regarding the direction of this association.

## 1 Introduction

Infertility affects approximately 10–15% of reproductive-aged couples worldwide and represents a major clinical and psychosocial challenge in gynecologic practice (1, 2). Among infertile couples, unexplained infertility remains one of the most common and clinically frustrating diagnostic categories, accounting for a substantial proportion of cases despite normal ovulatory function, tubal patency, and semen parameters (3, 4). In these women, the absence of an identifiable cause often leads to prolonged uncertainty, repeated treatment attempts, and significant emotional burden.

Women with unexplained infertility frequently report higher levels of anxiety, depressive symptoms, and perceived stress compared with fertile controls (5, 6). Beyond its psychological dimension, chronic stress is increasingly relevant in reproductive endocrinology because emotional stress may interact with neuroendocrine pathways regulating ovarian and reproductive function (7, 8).

The hypothalamic–pituitary–adrenal (HPA) axis is the principal endocrine system activated during stress. Activation of this pathway results in secretion of corticotropin-releasing hormone, adrenocorticotropic hormone, and cortisol. Persistent HPA axis activation has been proposed to interfere with the hypothalamic–pituitary–gonadal axis through altered gonadotropin-releasing hormone pulsatility, impaired luteinizing hormone dynamics, and changes in ovarian steroidogenesis (9, 10). These mechanisms may be particularly relevant in women with unexplained infertility, in whom subtle functional dysregulation may exist despite normal routine fertility evaluation.

Cortisol is the most commonly investigated biochemical marker of stress; however, its interpretation in infertility populations remains challenging because of circadian variation, pulsatile secretion, acute environmental influences, and interindividual variability (11, 12). However, single-time serum measurements may not adequately reflect chronic hypothalamic–pituitary–adrenal axis activity because cortisol secretion is influenced by circadian rhythm, acute environmental stressors, and interindividual variability. Dehydroepiandrosterone sulfate (DHEAS), an adrenal androgen with greater biological stability and less diurnal fluctuation, has been proposed as an alternative marker of chronic stress-system activity (13, 14). Nevertheless, evidence regarding the utility of cortisol and DHEAS in women with unexplained infertility remains limited and inconsistent.

In contrast, validated psychometric instruments may better capture the lived psychological burden experienced by patients undergoing infertility evaluation and treatment. The Hospital Anxiety and Depression Scale (HADS) is widely used in medical populations and allows separate assessment of anxiety and depressive symptoms while minimizing confounding by somatic complaints (15, 16). Elevated HADS scores in infertility populations have also been associated with reduced quality of life and treatment discontinuation (17, 18).

Although the relationship between infertility and psychological distress has been widely investigated, relatively limited data are available regarding the simultaneous evaluation of psychometric distress and single-time endocrine stress markers in women with unexplained infertility (19). Clarifying the relationship between psychometric distress and single-time endocrine stress markers may contribute to a better understanding of stress-related burden in women with unexplained infertility. Therefore, the present study aimed to investigate the association between psychological distress and endocrine stress markers, specifically cortisol and DHEAS, in women with unexplained infertility recruited from a tertiary gynecology clinic. We aimed to explore whether psychometric distress measures demonstrate a stronger association with unexplained infertility status than single-time peripheral endocrine measurements.

## 2 Materials and methods

### 2.1 Study design and population

This prospective case–control study was conducted between January and June 2021 at the gynecology outpatient clinic of a tertiary-care university-affiliated training and research hospital. Consecutive eligible women with primary unexplained infertility and age-matched fertile controls recruited from the same clinical setting were included during the study period. The study population consisted of women diagnosed with primary unexplained infertility and age-matched fertile controls.

The diagnosis of unexplained infertility was established by board-certified obstetrics and gynecology specialists in accordance with the diagnostic criteria of the American Society for Reproductive Medicine and the European Society of Human Reproduction and Embryology (ASRM/ESHRE) (4, 20). Unexplained infertility was defined as failure to achieve pregnancy after at least 12 months of regular unprotected intercourse in the presence of normal ovulatory function, bilateral tubal patency, and normal semen analysis.

A total of 107 women were initially screened. Five participants were excluded prior to enrollment because they did not meet predefined eligibility criteria or had incomplete clinical or laboratory data. After application of inclusion and exclusion criteria, 50 women with primary unexplained infertility and 52 fertile controls with at least one previous spontaneous pregnancy were included in the final analysis.

### 2.2 Inclusion and exclusion criteria

Women were eligible for inclusion if they were between 20 and 40 years of age and had regular ovulatory menstrual cycles. All participants underwent a standardized infertility evaluation, including pelvic ultrasonography, assessment of ovarian reserve (anti-Müllerian hormone, early follicular phase follicle-stimulating hormone and estradiol), thyroid-stimulating hormone, prolactin levels, hysterosalpingography or laparoscopy to confirm tubal patency, and semen analysis of the male partner according to World Health Organization criteria (21).

Exclusion criteria included polycystic ovary syndrome, premature ovarian insufficiency, endometriosis stage III–IV, uterine structural abnormalities, chronic systemic disease, psychiatric disorders requiring pharmacological treatment, use of hormonal or psychotropic medication within the preceding three months, smoking more than 10 cigarettes per day, night-shift work, and engagement in vigorous physical activity exceeding 150 minutes per week due to its potential impact on cortisol secretion (24).

Ovulatory function was confirmed using menstrual regularity, mid-luteal progesterone assessment, and ovarian reserve evaluation.

Although fertile controls were defined as women with at least one previous spontaneous pregnancy, additional variables including parity, time since last delivery, lactation status, hormonal contraceptive use, and prior infertility history were not systematically collected and were therefore not included in the adjusted analyses. This limitation has been acknowledged in the Discussion section.

### 2.3 Psychological assessment

Psychological distress was assessed using the Hospital Anxiety and Depression Scale (HADS), a self-report instrument specifically developed for use in medical populations (15). The HADS consists of two independent subscales assessing anxiety (HADS-A) and depression (HADS-D), each comprising seven items. Items are rated on a 4-point Likert scale ranging from 0 to 3, yielding subscale scores between 0 and 21.

Scores ≥8 on the HADS-Anxiety (HADS-A) and/or HADS-Depression (HADS-D) subscales were considered indicative of HADS-defined psychological distress, consistent with previous validation studies. The HADS instrument has demonstrated strong psychometric properties across diverse clinical settings and minimizes confounding by somatic symptoms that may overlap with medical conditions (16).

The Turkish version of the HADS has been validated by Aydemir et al., who reported good internal consistency and reliability (Cronbach’s α = 0.85), supporting its use in the present population (17). The HADS questionnaire was completed on the same day prior to blood sampling.

### 2.4 Biochemical measurements and sampling procedure

Venous blood samples were collected between 08:00 and 09:00 AM following overnight fasting to minimize circadian variability in hormone levels. All samples were obtained during the early follicular phase of the menstrual cycle (cycle days 2–5). Participants were instructed to avoid strenuous physical activity, caffeine intake, and acute stressors on the morning of blood sampling.

Serum cortisol and dehydroepiandrosterone sulfate (DHEAS) concentrations were measured as biochemical indicators of stress. Ovarian reserve markers, including follicle-stimulating hormone, estradiol, and anti-Müllerian hormone, were measured to confirm eligibility and exclude ovulatory dysfunction. Although factors such as time since awakening and stress related to venipuncture may influence cortisol levels, standardized timing and sampling conditions were applied to reduce acute variability (11, 19).

#### 2.4.1 Hormone assays

Serum cortisol and dehydroepiandrosterone sulfate (DHEAS) concentrations were quantified using an electrochemiluminescence immunoassay (ECLIA) method on the Roche Cobas 8000 modular analyzer series with Elecsys reagent kits (Roche Diagnostics, Mannheim, Germany), according to the manufacturer’s instructions All samples were analyzed in duplicate and processed within the same laboratory batch. The intra-assay coefficients of variation were <10% for both analytes.

### 2.5 Lifestyle factors and potential confounders

Data on sociodemographic characteristics, smoking status, physical activity, sleep patterns, and caffeine consumption were collected using structured questionnaires. These variables were evaluated as potential confounders due to their known associations with stress hormone regulation and psychological distress (6, 12).

### 2.6 Statistical analysis

Statistical analyses were performed using SPSS software (IBM SPSS Statistics for Windows, Version 23.0 (IBM Corp., Armonk, NY, USA)). Normality of continuous variables was assessed using the Kolmogorov–Smirnov test. Non-normally distributed continuous variables were analyzed using the Mann–Whitney U test, while normally distributed variables were compared using the independent samples t-test. Categorical variables were analyzed using the chi-square test or Fisher’s exact test, as appropriate. Multivariable logistic regression analysis was conducted to evaluate factors independently associated with unexplained infertility. Variables included in the final parsimonious adjusted model were age, body mass index, smoking status, residential environment, cortisol level, and HADS-defined psychological distress. Variables were selected based on clinical relevance, biological plausibility, and model stability considerations.

Given the modest sample size and the risk of model overfitting, the number of covariates included in the final adjusted model was intentionally restricted. DHEAS, follicle-stimulating hormone, and estradiol were explored during preliminary analyses but were not retained in the final parsimonious model because they did not substantially improve model performance and could increase instability of the estimated odds ratios. Results were expressed as odds ratios with 95% confidence intervals.

Exploratory receiver operating characteristic (ROC) curve analyses were performed to evaluate the discriminatory performance of cortisol and DHEAS levels for HADS-defined psychological distress. Exploratory non-parametric correlation analyses were additionally performed to evaluate potential associations between infertility duration and HADS-Anxiety score, HADS-Depression score, cortisol, and DHEAS levels. Statistical significance was defined as p < 0.05.

## 3 Results

### 3.1 Baseline sociodemographic and clinical characteristics

The baseline sociodemographic and clinical characteristics of the study population are summarized in **Table 1**. Women with unexplained infertility and fertile controls were comparable with respect to age, body mass index, smoking status, and residential environment, with no statistically significant differences observed between groups (*p* > 0.05 for all comparisons). These findings suggest that major demographic and lifestyle characteristics were generally comparable between groups. The mean duration of infertility among affected women was 3.4 ± 1.6 years (range: 1–7 years). None of the participants reported the use of hormonal therapy, psychotropic medication, or agents known to influence adrenal or ovarian hormone levels within the preceding three months.

**Table 1:** Baseline demographic and clinical characteristics of women with unexplained infertility and fertile controls.

Variable	Control Group (n=52)	Unexplained Infertility Group (n=50)	p value
Age (years)^a^	32.29 ± 6.26	30.92 ± 5.44	0.242
Body mass index (kg/m²)^a^	25.45 ± 3.75	24.94 ± 3.96	0.506
Smoking (cigarettes/day)^b^	0.00 (0–20)	0.00 (0–7)	0.317
Residence, n (%)^c^			0.918
Rural	14 (26.9)	13 (26.0)	
Urban	38 (73.1)	37 (74.0)	
Duration of infertility (years)^a^	—	3.4 ± 1.6	—

Data are presented as mean ± standard deviation or median (minimum–maximum), according to data distribution.

^a^ Independent samples t-test.

^b^ Mann–Whitney U test.

^c^ Chi-square test. Comparison of age, body mass index, smoking status, residential environment, and infertility duration between study groups. Continuous variables are presented as mean ± standard deviation or median (minimum–maximum), according to data distribution. Categorical variables are presented as number (%).

### 3.2 Psychological distress assessment

Psychological distress, assessed using the Hospital Anxiety and Depression Scale, was significantly more prevalent among women with unexplained infertility compared with fertile controls (**Table 2**). Median HADS-Anxiety scores were higher in the infertility group than in controls (10.0 vs. 6.0, *p* < 0.001). Similarly, median HADS-Depression scores were significantly elevated among infertile women (8.0 vs. 6.0, *p* = 0.020).

**Table 2:** Psychological distress profiles assessed by the hospital anxiety and depression scale (HADS) in women with unexplained infertility and fertile controls.

Variable	Control group (n=52)	Unexplained infertility Group (n=50)	p value
HADS-Anxiety score^a^	6.00 (2.00–15.00)	10.00 (1.00–20.00)	<0.001
HADS-Depression score^a^	6.00 (2.00–14.00)	8.00 (0.00–20.00)	0.020
HADS-defined psychological distress, n (%)^b^	22 (42.3)	38 (76.0)	0.001

Continuous variables are presented as median (minimum–maximum). Categorical variables are presented as number (%).

HADS-defined psychological distress was defined as HADS-Anxiety ≥8 and/or HADS-Depression ≥8.

^a^ Mann–Whitney U test.

^b^ Chi-square test. Comparison of HADS-Anxiety scores, HADS-Depression scores, and prevalence of HADS-defined psychological distress between groups. Continuous variables are presented as median (minimum–maximum). Categorical variables are presented as number (%). HADS-defined psychological distress was defined as HADS-Anxiety ≥8 and/or HADS-Depression ≥8.

Based on predefined HADS subscale cut-off values (HADS-A ≥8 and/or HADS-D ≥8), HADS-defined psychological distress was identified in 76% of women with unexplained infertility compared with 42.3% of fertile controls (p = 0.001). These results demonstrate a markedly higher emotional burden in women with unexplained infertility and support the clinical relevance of psychological screening in this population.

### 3.3 Hormonal measurements

Comparisons of endocrine and reproductive hormone parameters are presented in **Table 3**. Although follicle-stimulating hormone and estradiol levels were slightly lower in the infertility group than in controls (*p* = 0.026 and *p* < 0.001, respectively), all values remained within physiological reference ranges. Although these between-group differences reached statistical significance, their clinical relevance remains uncertain because all measured values remained within physiological reference ranges. No statistically significant differences were observed between groups with respect to serum cortisol, dehydroepiandrosterone sulfate, or anti-Müllerian hormone levels (*p* > 0.05 for all). Furthermore, correlation analyses revealed no significant associations between cortisol concentrations and gonadal hormones, suggesting independent regulation of adrenal and reproductive axes in this cohort.

**Table 3:** Reproductive and endocrine hormone parameters in women with unexplained infertility and fertile controls.

Variable	Control group (n=52) median (min–max)	Unexplained infertility group (n=50) median (min–max)	p value	Effect size *
Cortisol (µg/dL)^a^	10.53 (4.00–20.23)	13.52 (2.35–38.00)	0.060	0.178
DHEAS (µg/dL)^a^	147.40 (28.92–388.00)	138.75 (20.00–468.70)	0.274	-0.098
Follicle-stimulating hormone (mIU/mL)^a^	5.78 (2.45–11.50)	5.25 (2.24–10.50)	0.026	–
Estradiol (pg/mL)^a^	90.00 (25.00–450.00)	62.58 (20.25–96.30)	<0.001	–
Anti-Müllerian hormone (ng/mL)^a^	2.78 (0.22–5.54)	2.74 (2.00–10.37)	0.639	–

Continuous variables are presented as median (minimum–maximum). DHEAS: dehydroepiandrosterone sulfate.

^a^ Mann–Whitney U test.

*Effect sizes are provided for primary endocrine stress markers to facilitate interpretation of the magnitude and clinical relevance of between-group differences. Comparison of serum cortisol, dehydroepiandrosterone sulfate (DHEAS), follicle-stimulating hormone (FSH), estradiol (E2), and anti-Müllerian hormone (AMH) levels between groups. Continuous variables are presented as median (minimum–maximum). Effect sizes for primary endocrine stress markers are additionally presented to facilitate interpretation of the magnitude of between-group differences.

Although cortisol levels tended to be numerically higher in women with unexplained infertility, the observed effect sizes for cortisol and DHEAS comparisons were small, supporting limited discriminatory capacity of isolated single-time endocrine measurements in this cohort.

Although serum cortisol levels showed a non-significant trend toward higher values in women with unexplained infertility (p = 0.060), this finding should be interpreted cautiously given the small effect size and the inherent variability of single-time cortisol measurements.

### 3.4 Logistic regression analysis

To evaluate independent factors associated with unexplained infertility, both unadjusted and adjusted logistic regression models were constructed (**Table 3**).

In univariate analysis, age, body mass index, smoking status, cortisol, and DHEAS were not significantly associated with infertility status. In contrast, HADS-defined psychological distress remained significantly associated with unexplained infertility status (odds ratio [OR] 4.318, 95% confidence interval [CI] 1.844–10.111, *p* = 0.001).

In the final parsimonious multivariable model adjusted for age, body mass index, smoking status, residential environment, cortisol level, and HADS-defined psychological distress, psychological distress remained the only independently associated factor with unexplained infertility (adjusted OR 3.907, 95% CI 1.610–9.478, p = 0.003) (**Table 4**). Cortisol did not demonstrate an independent association after adjustment. DHEAS, follicle-stimulating hormone, and estradiol were explored during preliminary analyses but were not retained in the final parsimonious model because they did not substantially improve model performance and could increase instability related to model overfitting in the setting of a modest sample size.

**Table 4:** Parsimonious multivariable logistic regression model evaluating factors independently associated with unexplained infertility status.

Variable	Adjusted OR	95% CI	p value
Age	0.981	0.912–1.055	0.605
Body mass index	0.985	0.883–1.099	0.788
Smoking	0.695	0.189–2.550	0.583
Living area	1.086	0.414–2.850	0.867
Cortisol	1.029	0.949–1.115	0.489
HADS-defined psychological distress	3.907	1.610–9.478	0.003

Adjusted odds ratios were derived from a parsimonious multivariable logistic regression model. Variables included in the final model were selected based on clinical relevance, biological plausibility, and model stability considerations to reduce the risk of overfitting given the modest sample size.

HADS-defined psychological distress was defined as HADS-Anxiety ≥8 and/or HADS-Depression ≥8. Adjusted odds ratios (ORs), 95% confidence intervals (CIs), and p values for variables included in the final parsimonious multivariable model, including age, body mass index, smoking status, residential environment, cortisol, and HADS-defined psychological distress.

### 3.5 Receiver operating characteristic analysis

**Figure 1:**
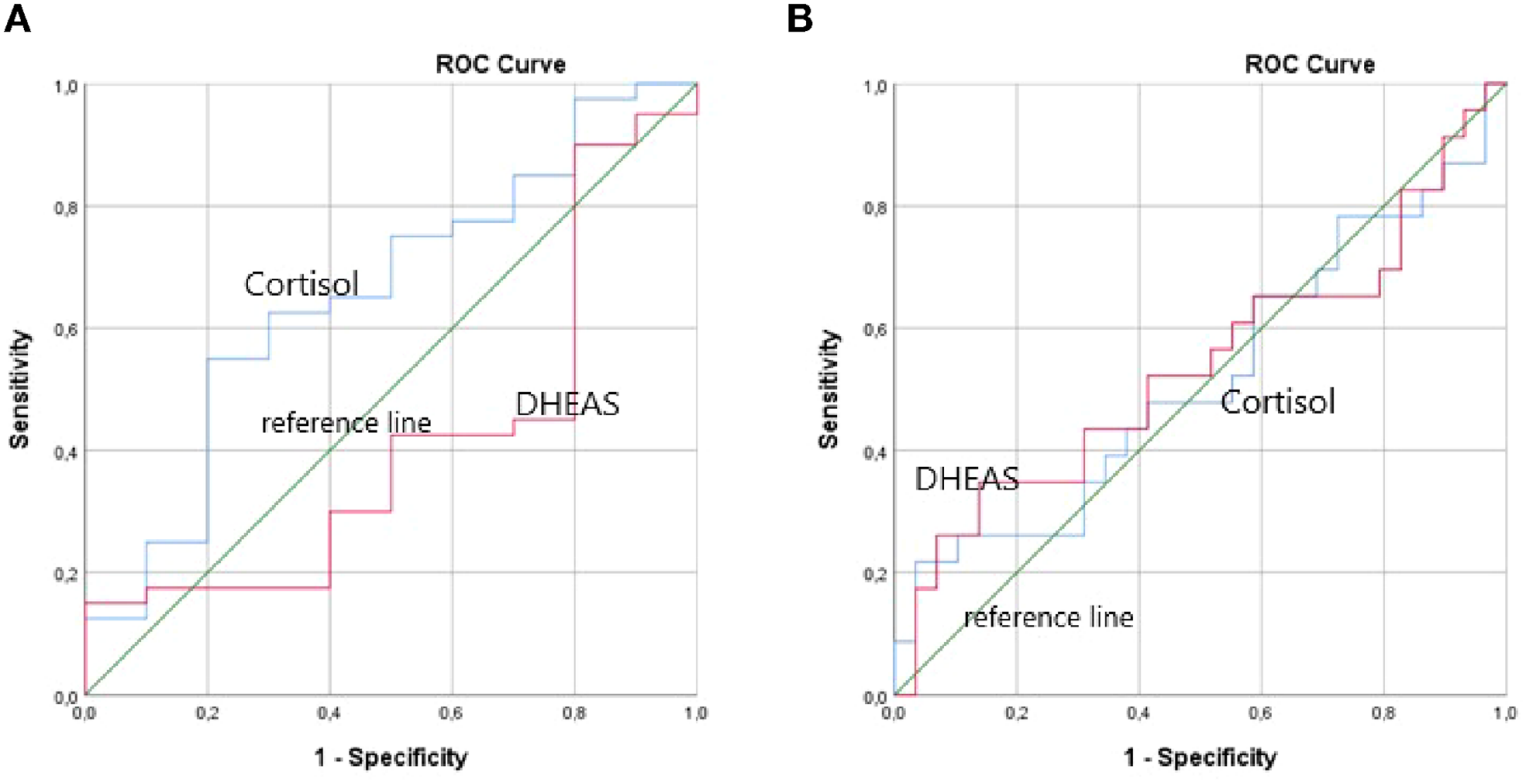
ROC curves of patient and control group. Exploratory receiver operating characteristic (ROC) curve analyses evaluating the discriminatory performance of single-time serum cortisol and DHEAS levels for HADS-defined psychological distress. **(A)** Women with unexplained infertility. **(B)** Fertile controls. Area under the curve (AUC) values demonstrated limited discriminatory performance of single-time endocrine stress markers for identifying clinically relevant psychological distress in both groups.

Exploratory receiver operating characteristic (ROC) curve analyses were performed to evaluate the discriminatory performance of cortisol and DHEAS levels for HADS-defined psychological distress (**Figure 1**). In women with unexplained infertility, cortisol yielded an area under the curve (AUC) of 0.655 (95% CI 0.464–0.846, *p* = 0.133), while DHEAS demonstrated an AUC of 0.587 (95% CI 0.388–0.787, *p* = 0.396). Comparable results were observed in the control group, with AUC values approximating 0.5 and non-significant *p* values.

Collectively, these findings suggest that isolated single-time cortisol and DHEAS measurements demonstrate limited discriminatory capacity for identifying HADS-defined psychological distress in this cohort.

Exploratory non-parametric correlation analyses demonstrated no significant correlations between infertility duration and HADS-Anxiety score, HADS-Depression score, cortisol level, or DHEAS concentration (all p > 0.05).

## 4 Discussion

The present study examined the relationship between psychological distress and endocrine stress markers in women with unexplained infertility, with particular emphasis on comparing the relative value of psychometric assessment and biochemical indicators. Our findings demonstrate that psychological distress assessed using the Hospital Anxiety and Depression Scale (HADS) was significantly more prevalent among women with unexplained infertility, whereas serum cortisol and dehydroepiandrosterone sulfate (DHEAS) levels did not differ between infertile and fertile women and showed no meaningful discriminatory capacity. These findings suggest that psychometric assessment may better reflect perceived emotional burden than isolated single-time biochemical measurements in women with unexplained infertility.

### 4.1 Psychological distress and unexplained infertility

A central finding of this study is the strong and independent association between HADS-defined psychological distress and unexplained infertility. Even after adjustment for age, body mass index, smoking status, residential environment, cortisol level, and HADS-defined psychological distress, psychological distress remained independently associated with infertility status. This observation is consistent with previous evidence demonstrating a close relationship between infertility experience and psychological distress (1, 2).

Women diagnosed with unexplained infertility often face prolonged diagnostic uncertainty and repeated unsuccessful attempts to conceive, which may intensify feelings of helplessness, anxiety, and depressive symptoms. The absence of an identifiable organic cause may further increase emotional burden among women undergoing infertility evaluation (8).

### 4.2 Reproductive hormones and neuroendocrine adaptation

Although follicle-stimulating hormone and estradiol levels were modestly lower in the infertility group, all values remained within physiological reference ranges. Previous studies have suggested potential interactions between psychological stress and hypothalamic–pituitary–gonadal axis regulation; however, the clinical significance of subtle hormonal variations within physiological ranges remains uncertain (22, 23). Importantly, no significant correlations were detected between cortisol concentrations and reproductive hormones in the present study, suggesting differential regulation of adrenal and gonadal axes under chronic stress conditions (11).

### 4.3 Limited utility of biochemical stress markers

The absence of significant differences in serum cortisol and DHEAS levels between infertile and fertile women aligns with growing evidence highlighting the limitations of single-time biochemical stress measurements. Serum cortisol is subject to pronounced circadian and ultradian variability and is influenced by multiple acute factors, including sleep quality, physical activity, nutritional status, and stress related to venipuncture itself (12, 24). Consequently, isolated morning cortisol measurements may reflect transient physiological states rather than cumulative psychological stress.

Although DHEAS demonstrates greater stability than cortisol, its role as a reliable marker of chronic psychological stress in infertility remains insufficiently defined. Recent methodological and clinical studies suggest that more stable biomarkers, such as hair cortisol concentration, may provide a more reliable assessment of long-term hypothalamic–pituitary–adrenal axis activity and cumulative stress exposure (25, 26). In reproductive medicine, elevated hair cortisol levels have been associated with poorer assisted reproductive technology outcomes, underscoring the relevance of chronic stress assessment (26).

Our findings are consistent with experimental evidence demonstrating that stress-related endocrine responses exhibit substantial interindividual variability across the menstrual cycle, limiting the interpretability of isolated cortisol measurements. Maki et al. reported significant cycle-dependent differences in cortisol responsivity following psychosocial stress, emphasizing that single-point peripheral hormone levels may not reliably reflect emotional stress burden (27).

Prolactin has also been recognized as a neuroendocrine mediator linking psychological stress and reproductive dysfunction. Even subtle variations in prolactin levels within physiological ranges may influence hypothalamic–pituitary–gonadal axis regulation and fertility outcomes (28, 30). Furthermore, DHEA and DHEAS have been evaluated as biomarkers of stress, although systematic reviews report heterogeneous findings and limited reliability for assessing chronic psychological burden (14). Similar associations between psychosocial stress, reproductive dysfunction, and gynecologic disorders have also been described in women with endometriosis, emphasizing the broader clinical relevance of psychological assessment in reproductive medicine (29, 31).

### 4.4 Clinical value of psychometric assessment

In contrast to biochemical markers, validated psychometric instruments offer direct insight into the subjective experience of stress. The HADS was specifically developed to minimize confounding by somatic symptoms and allows independent assessment of anxiety and depressive domains. In infertility populations, elevated HADS scores have been associated with reduced quality of life, impaired treatment adherence, and increased likelihood of treatment discontinuation (18, 19).

In the present study, HADS effectively discriminated between infertile and fertile women and demonstrated robust associations across unadjusted and adjusted analytical models. These findings support the integration of routine psychological screening into infertility evaluation and emphasize the importance of addressing emotional well-being as part of comprehensive infertility care.

### 4.5 Integrative perspective and clinical implications

A major strength of this study lies in its integrative design, combining standardized psychometric assessment with synchronized hormonal measurements. Contemporary stress research increasingly supports multidimensional models that integrate psychological and biological indicators to better capture stress-related vulnerability (28). Our findings suggest that psychometric distress measures may provide complementary information regarding emotional burden in women with unexplained infertility. From a clinical perspective, psychological assessment may help identify emotional burden in women undergoing infertility evaluation and may support a more holistic approach to patient care.

### 4.6 Limitations and future directions

Several limitations should be acknowledged. The relatively modest sample size may have limited statistical power, particularly for detecting subtle hormonal differences between groups. Although cortisol sampling was standardized, single-time measurements cannot adequately reflect long-term stress exposure. Single-time serum measurements of cortisol and DHEAS may not adequately reflect chronic stress exposure, and repeated or alternative measures such as hair or salivary cortisol may provide more reliable assessment of long-term neuroendocrine activity. The cross-sectional design precludes causal inference regarding the directionality of the relationship between psychological distress and infertility. Due to the cross-sectional design, psychological distress may represent either a contributing factor to infertility or a consequence of prolonged infertility experience; therefore, causal interpretations should be avoided.

In addition, couple-level psychological and relational factors, socioeconomic determinants, and treatment-related stressors were not comprehensively evaluated and may have contributed to residual confounding.

Future studies should employ longitudinal and multimodal designs incorporating repeated hormonal sampling, hair cortisol concentration, and diurnal salivary cortisol profiles to improve characterization of chronic stress. Integrative psychometric–biological models may provide a more comprehensive understanding of the stress–infertility interface and enhance translational relevance in reproductive medicine (28, 31).

Although prolactin levels were measured during screening to exclude hyperprolactinemia, all values were within normal reference ranges and therefore were not included in the statistical analysis. Nevertheless, prolactin may act as a neuroendocrine mediator linking psychological stress and reproductive function, and future studies incorporating subtle variations in prolactin levels may provide additional insights.

## 5 Conclusion

The findings of the present study suggest that psychological distress is more prevalent among women with unexplained infertility, whereas single-time serum cortisol and dehydroepiandrosterone sulfate measurements were not significantly different between groups. These results highlight the limited utility of single-time endocrine measurements in capturing chronic psychological burden in women with unexplained infertility.

By simultaneously evaluating psychometric distress and synchronized endocrine measurements within the same cohort, this study contributes to the growing literature investigating stress-related factors in unexplained infertility. Psychological distress remained significantly associated with unexplained infertility status after adjustment for relevant confounders; however, the cross-sectional case–control design does not permit causal or directional inference. These findings support the relevance of psychological assessment in women undergoing infertility evaluation and highlight the importance of considering emotional well-being during infertility care. Single-time endocrine measurements alone may not adequately reflect the complex psychological and neuroendocrine dimensions of infertility-related stress. Future longitudinal and multimodal studies are needed to better clarify the temporal and biological relationship between psychological distress and reproductive outcomes in women with unexplained infertility.

## Data Availability

The raw data supporting the conclusions of this article will be made available by the authors, without undue reservation.

## References

[1] Greil AL Slauson-Blevins K McQuillan J . The experience of infertility: a review of recent literature. Soc Sci Med. (2010) 71:9–15. doi: 10.1016/j.socscimed.2010.08.006 20003036 PMC3383794

[2] Rooney KL Domar AD . The impact of stress on fertility. Dialogues Clin Neurosci. (2018) 20:41–7. doi: 10.1097/gco.0000000000000261 29946210 PMC6016043

[3] Practice Committee of the American Society for Reproductive Medicine . Diagnostic evaluation of the infertile female: a committee opinion. Fertil Steril. (2015) 103:e44–50. doi: 10.1016/j.fertnstert.2015.03.019 25936238

[4] Crosignani PG Rubin BL . Optimal use of infertility diagnostic tests and treatments. The ESHRE Capri Workshop Group. Hum Reprod. (2000) 15:723–32. doi: 10.1093/humrep/15.3.723 10686227

[5] Cousineau TM Domar AD . Psychological impact of infertility. Best Pract Res Clin Obstet Gynaecol. (2007) 21:293–308. doi: 10.1016/j.bpobgyn.2006.12.003 17241818

[6] Frederiksen Y Farver-Vestergaard I Skovgård NG Ingerslev HJ Zachariae R Boivin J . Psychological distress and fertility outcomes. Hum Reprod. (2015) 30:111–22. doi: 10.1093/humrep/deu284 25355589 PMC4262467

[7] Chrousos GP . Stress and disorders of the stress system. Nat Rev Endocrinol. (2009) 5:374–81. doi: 10.1038/nrendo.2009.106 19488073

[8] Berga SL Loucks TL Cameron JL . Endocrine and metabolic responses to stress in women. J Clin Endocrinol Metab. (2001) 86:2590–7. doi: 10.1210/jcem.86.6.7617 31642201

[9] Rivier C Rivest S . Effect of stress on the activity of the hypothalamic–pituitary–gonadal axis. Biol Reprod. (1991) 44:915–23. doi: 10.1095/biolreprod44.6.915 1661182

[10] Viau V . Functional cross-talk between the hypothalamic-pituitary-gonadal and -adrenal axes. J Neuroendocrinol. (2002) 14:506–13. doi: 10.1046/j.1365-2826.2002.00798.x 12047726

[11] Adam EK Kumari M . Assessing cortisol in epidemiological research. Psychoneuroendocrinology. (2009) 34:1423–36. doi: 10.1016/j.psyneuen.2009.06.011 19647372

[12] Fries E Dettenborn L Kirschbaum C . The cortisol awakening response (CAR): facts and future directions. Int J Psychophysiol. (2009) 72:67–73. doi: 10.1016/j.ijpsycho.2008.03.014 18854200

[13] Izawa S Sugaya N Shirotsuki K Yamada KC Ogawa N Ouchi Y . Salivary dehydroepiandrosterone secretion in response to acute psychosocial stress and its correlations with biological and psychological changes. Biol Psychol. (2008) 79:294–8. doi: 10.1016/j.biopsycho.2008.07.003 18706968

[14] Dutheil F de Saint Vincent S Pereira B Schmidt J Moustafa F Charkhabi M . DHEA as a biomarker of stress: a systematic review and meta-analysis. Front Psychiatry. (2021) 12:688367. doi: 10.3389/fpsyt.2021.688367 34295276 PMC8290065

[15] Zigmond AS Snaith RP . The hospital anxiety and depression scale. Acta Psychiatr Scand. (1983) 67:361–70. doi: 10.1111/j.1600-0447.1983.tb09716.x 6880820

[16] Bjelland I Dahl AA Haug TT Neckelmann D . The validity of the HADS: an updated literature review. J Psychosom Res. (2002) 52:69–77. doi: 10.1016/S0022-3999(02)00447-8 11832252

[17] Ayemir Ö Güvenir T Küey L Kültür S . Hastane Anksiyete ve Depresyon Ölçeği Türkçe formunun geçerlilik ve güvenilirliği. Türk Psikiyatr Der. (1998) 9:128–37.

[18] Matthiesen SM Frederiksen Y Ingerslev HJ Zachariae R . Stress and ART outcomes. Hum Reprod. (2011) 26:2763–76. doi: 10.1093/humrep/der246 21807816

[19] Slavich GM Shields GS . Integrative psychological and biological approaches to stress. Annu Rev Clin Psychol. (2023) 19:1–28. doi: 10.1146/annurev-clinpsy-072720-014809 37159285

[20] World Health Organization . WHO laboratory manual for the examination and processing of human semen. 5th ed. Geneva: WHO (2010).

[21] Kudielka BM Kirschbaum C . Sex differences in HPA axis responses to stress. Psychoneuroendocrinology. (2005) 30:1–15. doi: 10.1016/j.psyneuen.2004.08.009 15740829

[22] Viau V . Functional cross-talk between the hypothalamic–pituitary–gonadal and adrenal axes. J Neuroendocrinol. (2002) 14:506–13. doi: 10.1046/j.1365-2826.2002.00899.x 12047726

[23] Roney JR Simmons ZL . Hormonal predictors of female reproductive physiology and behavior. Horm Behav. (2013) 63:636–45. doi: 10.1016/j.yhbeh.2013.02.013 23601091

[24] Stalder T Steudte-Schmiedgen S Kirschbaum C Alexander N Klucken T Miller R . Assessment of cortisol in human hair: updated methodological considerations and clinical applications. Psychoneuroendocrinology. (2022) 145:105889. doi: 10.1016/j.psyneuen.2022.105889 35944454

[25] Stalder T Kirschbaum C . Hair cortisol analysis: state of the art and future directions. Brain Behav Immun. (2012) 26:1019–29. doi: 10.1016/j.bbi.2012.02.002 22366690

[26] Khoury JE Bosquet Enlow M Plamondon A Lyons-Ruth L . Hair cortisol concentration predicts outcomes of assisted reproductive technology. Psychoneuroendocrinology. (2019) 103:104–17. doi: 10.1016/j.psyneuen.2019.01.009 30682626 PMC6450779

[27] Maki PM Mordecai KL Rubin LH Sundermann E Savarese A Eatough E . Menstrual cycle effects on cortisol responsivity and emotional retrieval following a psychosocial stressor. Horm Behav. (2015) 74:201–8. doi: 10.1016/j.yhbeh.2015.06.023 26187711 PMC4876953

[28] Lewiński A Brzozowska MM . Hyperprolactinemia and reproductive dysfunction—contemporary perspectives. Gynecol. Reprod. Endocrinol. Metabol. (2022) 3:94–8.

[29] Fiala L Lenz J Bob P . Effect of psychosocial trauma and stress on sexual dysfunction in women with endometriosis. Med (Baltimore). (2021) 100:e26836. doi: 10.1097/MD.0000000000026836 34397850 PMC8341311

[30] Faron-Górecka A Szlachta M Kolasa M Solich J Górecki A . The involvement of prolactin in stress-related disorders. Int J Environ Res Public Health. (2023) 20:3257. doi: 10.3390/ijerph20043257 36833950 PMC9959798

[31] Rodríguez-Lozano DC Meza-Rodríguez MDP Cruz-Orozco OP Sánchez-Ramírez B Olguin-Ortega A Silvestri-Tomassoni JR . Emotional dysregulation in women with endometriosis with cyclical and non-cyclical chronic pelvic pain. BMC Womens Health. (2022) 22:525. doi: 10.1186/s12905-022-02066-5 36526995 PMC9758838

